# Screening and Genomic Profiling of Antimicrobial Bacteria Sourced from Poultry Slaughterhouse Effluents: Bacteriocin Production and Safety Evaluation

**DOI:** 10.3390/genes15121564

**Published:** 2024-12-02

**Authors:** Nuria Peña, Irene Lafuente, Ester Sevillano, Javier Feito, Diogo Contente, Estefanía Muñoz-Atienza, Luis M. Cintas, Pablo E. Hernández, Juan Borrero

**Affiliations:** Departamento de Nutrición y Ciencia de los Alimentos (NUTRYCIAL), Sección Departamental de Nutrición y Ciencia de los Alimentos (SD-NUTRYCIAL), Facultad de Veterinaria, Universidad Complutense de Madrid (UCM), Avenida Puerta de Hierro, s/n, 28040 Madrid, Spain; nuriapen@ucm.es (N.P.); irelafue@ucm.es (I.L.); estsev01@ucm.es (E.S.); j.feito@ucm.es (J.F.); diogodas@ucm.es (D.C.); ematienza@ucm.es (E.M.-A.); lcintas@vet.ucm.es (L.M.C.); ehernan@vet.ucm.es (P.E.H.)

**Keywords:** bacteriocins, *Enterococcus faecium*, enterocins, *Lactococcus lactis*, *Lactococcus garvieae*, lactococcins, *E. coli*, colicin, microcin

## Abstract

**Background/Objectives:** Antimicrobial-resistant (AMR) pathogens represent a serious threat to public health, particularly in food production systems where antibiotic use remains widespread. As a result, alternative antimicrobial treatments to antibiotics are essential for effectively managing bacterial infections. This study aimed to identify and characterize novel antimicrobial peptides produced by bacteria, known as bacteriocins, as well as to recognize safe bacteriocin-producing strains, sourced from poultry slaughterhouse effluents. **Methods:** A total of 864 bacterial isolates were collected across eight stages of a poultry slaughter line and screened for antimicrobial activity against Gram-positive and Gram-negative indicator strains. Whole-genome sequencing (WGS) was performed on 12 selected strains, including *Enterococcus faecium* (6 isolates)*, Lactococcus lactis* (1 isolate), *Lactococcus garvieae* (1 isolate) and *Escherichia coli* (4 isolates). The presence of bacteriocin gene clusters (BGC), antibiotic resistance genes (ARG), and virulence factors (VF) was analyzed. The antimicrobial activity of a novel bacteriocin was further evaluated using in vitro cell-free protein synthesis (IV-CFPS). **Results:** WGS revealed multiple BGCs, including a novel class IId bacteriocin, lactococcin P1A (LcnP1A), in *L. lactis* SWD9. LcnP1A showed antimicrobial activity against various indicator strains, including *Listeria monocytogenes*. While most bacteriocin-encoding strains harbored ARGs and VFs, *E. faecium* SWG6 was notable for its absence of ARGs and minimal VFs, highlighting its potential as a probiotic. **Conclusions**: These findings underscore the importance of discovering novel bacteriocins and safer bacteriocin producing strains to address antimicrobial resistance in the food chain. Further research would validate the efficacy of both the novel lactococcin P1A bacteriocin and the *E. faecium* SWG6 isolate for application in processed food and animal production systems.

## 1. Introduction

The global rise of antimicrobial resistance (AMR) has become one of the most pressing public health challenges of the 21st century. As antibiotics continue to be heavily used in both human and veterinary medicine, their overuse and misuse have accelerated the emergence of antibiotic-resistant bacteria [1]. This is especially problematic in the agricultural sector, where antibiotics are extensively used for disease prevention, growth promotion, and infection control in livestock [2]. The widespread use of antibiotics in these settings has resulted in the accumulation of resistant bacteria in the food production chain, raising concerns about the transmission of AMR to humans through consumption or environmental exposure [3]. A recent report estimates that, without immediate intervention, global antibiotic consumption could increase by 200% by 2030, further exacerbating the AMR crisis [4].

Poultry production is a key area of focus for AMR control due to the high levels of antibiotic use and the potential for cross-contamination between livestock and humans [5,6]. Effluents from poultry slaughterhouses, in particular, are significant reservoirs of antibiotic-resistant bacteria, providing an environment in which bacteria can acquire and exchange resistance genes. These effluents, which can enter the environment via water and soil systems, pose a serious risk for the spread of AMR through indirect pathways. This includes the contamination of crops irrigated with tainted water and the introduction of resistant bacteria into the wider food chain [7]. Controlling the spread of pathogenic bacteria in these settings requires interventions that not only reduce bacterial presence but also prevent the dissemination of resistance genes. To combat this growing threat, there is an urgent need for innovative antimicrobial strategies that can be safely integrated into the food production systems. These strategies should reduce reliance on conventional antibiotics while controlling pathogenic bacteria [8].

Among the various strategies being explored, bacteriocins have emerged as a promising alternative to traditional antibiotics. Bacteriocins are ribosomally synthesized antimicrobial peptides produced by bacteria, exhibiting potent activity against closely related bacterial strains. Unlike broad-spectrum antibiotics, bacteriocins typically have a narrow spectrum of activity, allowing them to target specific pathogens without disrupting the entire microbial community [9]. This characteristic makes bacteriocins especially appealing for food production, as they play a crucial role in maintaining a healthy balance of beneficial bacteria, which is essential for ensuring both food safety and quality. Additionally, bacteriocins are biodegradable, non-toxic, and less likely to induce resistance in target organisms, making them ideal candidates for tackling antimicrobial resistance (AMR) in a sustainable way [10,11]. Numerous studies have highlighted the potential of bacteriocins to inhibit foodborne pathogens such as *L. monocytogenes*, *Clostridium perfringens*, and *Salmonella* spp., which are commonly associated with poultry and other livestock products [12,13,14].

In addition to investigating bacteriocins, the use of probiotics has emerged as an effective strategy to combat pathogenic bacteria and improve animal health in livestock production [15]. Probiotics, which are live microorganisms that confer health benefits to the host when administered in adequate amounts, have gained significant attention in recent years for their ability to enhance gut health, boost immune function, and reduce the need for antibiotics in animal farming [16,17]. Several strains of lactic acid bacteria (LAB), including strains of *E. faecium* and *Lactococcus* species, are already commercially available as probiotics for poultry and other livestock [18,19]. For instance, *E. faecium* SF68 has been extensively studied and is widely used in both human and veterinary applications due to its ability to improve gut flora balance and prevent gastrointestinal infections. Recent studies have shown the potential of certain LAB in controlling the proliferation of *Salmonella typhimurium* and mitigating intestinal barrier damage caused by necrotic enteritis in broiler chickens [20,21,22,23]. By fostering a competitive environment and producing antimicrobial compounds, these probiotics effectively inhibit the growth of pathogens, reducing the incidence of infections and the subsequent need for antibiotic treatments [24,25]. This dual role of probiotics in promoting health and preventing pathogen growth makes them an essential tool in the fight against AMR, particularly within food production systems.

This study sought to identify novel bacteriocins and bacteriocin-producing strains with antimicrobial activity derived from poultry slaughterhouse effluents. By isolating both Gram-positive and Gram-negative bacteria, we targeted a broad spectrum of potential bacteriocin producers capable of inhibiting foodborne pathogens. Samples were collected from various stages of the slaughterhouse process to ensure a diverse range of bacterial communities. The isolates were then screened for antimicrobial activity against Gram-positive and Gram-negative indicator strains to identify those exhibiting antimicrobial properties. The whole-genome sequencing (WGS) of the most active antimicrobial isolates may reveal the occurrence of multiple bacteriocin gene clusters (BGC) within their genomes. The synthesis and production of putative mature bacteriocins using an in vitro cell-free protein synthesis (IV-CFPS) approach, implemented by our research group, will facilitate the identification of the most promising circular and class II bacteriocins. A combination of phenotypic tests and in silico genomic profiling of the selected bacteriocin-producing strains will assess their safety as potential probiotics.

## 2. Materials and Methods

### 2.1. Sampling Sites and Sample Treatment

Sample acquisition was conducted at a Spanish poultry slaughterhouse with a slaughter capacity of >28.000 tons of chicken per year. Sampling of process-water and wastewater was performed between October and November 2021. Overall, 8 water samples were collected for this study, including those used from the cleaning of the chicken transport truck (TT); the cleaning of the empty cages following passage of the chickens into the processing line (EC); the drains during the stunning (ST), scalding (SC), de-feathering (DF), evisceration (EV) and washing of the carcasses (WC); and the general sewage water prior to treatment by the facility (SW). At each sampling site, 50 mL water samples were collected in sterile 50 mL centrifuge tubes (Thermo Fisher Scientific, Waltham, MA, USA) and kept immediately on ice until further processing. Once in the laboratory, 1 mL of each water sample was mixed with 9 mL of Ringer’s solution (Oxoid, Basingstoke, UK) to make an initial 10^−1^ dilution. Subsequent ten-fold serial dilutions were performed using Ringer’s solution, and 100 μL of each dilution was plated onto: (a) Brain Heart Infusion (BHI) (Oxoid) agar (Scharlab, Barcelona, Spain) (1.5% *w*/*v*) plates; which is a non-selective medium, (b) MacConkey plates (Biomerieux, Marcy-l’Étoile, France), which selects for Gram-negative enteric bacteria; and (c) Salmonella–Shigella (SS) and (d) Hektoen plates (Biomerieux), which select for *Salmonella* spp. and some strains of *Shigella* spp. Plates were incubated at 37 °C aerobically until colonies were visible (usually after 48–72 h). Colonies exhibiting distinct morphologies were carefully selected and inoculated into 250 μL aliquots of BHI (colonies obtained from BHI plates) broth or LB (Scharlab) (colonies obtained from MacConkey, SS and Hektoen plates) broth in 96-well plates (Nunc, Roskilde, Denmark). Plates were grown at 37 °C aerobically for 48 h and stored at −80 °C with 20% glycerol (Sigma-Aldrich, St. Louis, MO, USA) for further analysis.

### 2.2. Screening of Isolates with Antimicrobial Activity Against Gram-Positive Indicators

The antimicrobial activity of selected isolates against different Gram-positive indicators was evaluated using a stamp-on-agar test (STOAT). Briefly, BHI-derived isolates grown in 96-well plates and stored at −80 °C were transferred by using a 96-pin microplate replicator (Boekel Scientific, Philadelphia, PA, USA) to 96-well plates with 250 μL of BHI per well. Plates were incubated aerobically at 37 °C for 48 h. Then, 2.5 µL of the grown cultures were seeded using a 48-pin microplate replicator (Boekel Scientific) into BHI 1.5% agar plates previously overlayed with 5 mL BHI 0.8% agar seeded with 10^5^ to 10^6^ CFU/mL of an overnight culture of *Pediococcus damnosus* CECT 4797. Plates were incubated at 30 °C for 24 h, after which zones of inhibition surrounding the colony spots were measured. The most active strains were re-evaluated for antimicrobial activity using the STOAT against the indicator strains *P. damnosus* CECT 4797, *L. monocytogenes* CECT 4032, *C. perfringens* DICM15/00067-5A, and *Staphylococcus aureus* ZTA11/00310ST. Plates were incubated at 30 °C for 24 h, after which zones of inhibition surrounding the spots were measured as the diameter of the halos of inhibition in millimeters.

### 2.3. Screening of Isolates with Antimicrobial Activity Against Gram-Negative Indicators

The antimicrobial activity of selected isolates against different Gram-negative indicators was evaluated using the STOAT as described above, with some minor modifications. LB-derived isolates grown in 96-well plates and stored at −80 °C were transferred using a 96-pin microplate replicator to 96-well plates with 100 μL of M9 minimal media (12.8 g/L Na_2_HPO_4_ x7H_2_O, 3 g/L KH_2_PO_4_, 0.5 g/L NaCl, 1 g/L NH_4_Cl, 2 mM MgSO_4_, 0.1 mM CaCl_2_, 0.4% [p/v] 0.5 mM thiamine, and 8 g/L D-glucose) per well. Plates were incubated aerobically at 37 °C for 3 h, and then 100 µL M9 media supplemented with 0.25 µg/mL of mytomicin C (Sigma) was added to each well and the plates were incubated aerobically at 37 °C for another 2 h. A 48-pin microplate replicator was used to stamp 2.5 µL spots from the 96-well plates onto M9 1.5% agar plates previously overlayed with 5 mL of M9 0.8% agar seeded with 10^5^ to 10^6^ CFU/mL of an overnight culture of *E. coli* DH5α. Plates were incubated at 37 °C for 24 h, after which zones of inhibition surrounding the colonies were measured. The most active strains were re-evaluated for antimicrobial activity using the STOAT against four different *E. coli* indicator strains.

### 2.4. Taxonomic Identification of the Bacterial Isolates

Thirteen isolates with antimicrobial activity against Gram-positive indicators and six isolates showing antimicrobial activity against Gram-negative indicators were initially taxonomically identified by PCR amplification and sequencing of 16S rRNA (16S rDNA) sequences. The InstaGene^TM^ matrix (BioRad, Hercules, CA, USA) resin was used for extraction and purification of the genomic DNA. The isolated DNA was further used as a template to amplify a variable region of the 16S rRNA gene using primers rD1 (5′ TAA GGA GGT GAT CCA GCC 3′) and fD1 (5′ AGA GTT TGA TCC TGG CTC AG 3′) (Thermo Fisher Scientific). PCR products were purified with the NucleoSpinR Gel and PCR Clean-up (Macherey-Nagel, Düren, Germany) and subjected to Sanger sequencing (Eurofins Genomics, Ebersberg, Germany). To determine the corresponding species identity, a comparative sequence analysis (BLASTn) was performed against available sequence data in the National Center for Biotechnology Information (NCBI) database.

### 2.5. Random Amplified Polymorphic DNA (RAPD) Technique to Analyze the Diversity of the Bacterial Isolates

Isolates of the same genus were further evaluated to analyze their inter- and intra-specific genetic variations by using the random amplified polymorphic DNA (RAPD) technique. Briefly, DNAs from all isolates (19 in total) were used as templates in PCR reactions with primer OPL5 (5′-ACGCAGGCAC-3′) and the Dream Taq Green PCR Master Mix (2x) (Thermo Fisher Scientific), as previously described [26]. The resulting amplification products were run at 90 V for 60 min in an electrophoresis chamber (BioRad), and the visualization of the band patterns was performed in a ChemiDoc Imaging System (BioRad) with HyperLadder 100 bp (Bioline, Cincinnati, OH, USA) as a molecular weight marker.

### 2.6. Evaluation of the Antimicrobial Activity of Selected Isolates Versus a Larger Panel of Indicators

Cell-free supernatants (CFS) of the isolates with the highest antimicrobial activity against Gram-positive indicators (isolates producing halos of inhibition larger than 1 mm or antimicrobial activity against at least two of the Gram-positive indicator strains evaluated) were evaluated for their antimicrobial activity by using an agar diffusion test (ADT) [27] against *P. damnosus* CECT 4797, *C. perfringens* CECT 4110, *L. monocytogenes* CECT 4032, *L. grayii* CECT 931, *L. seeligeri* CECT 917, *Streptococcus suis* C2969/03, 4 strains of *E. faecium* resistant to vancomycin (VRE), 2 strains of *E. faecalis*, 4 strains of *L. lactis*, 1 strain of *L. garvieae*, and 1 strain of *S. aureus*. CFS were obtained by centrifugation of overnight cultures of the selected isolates at 13,000 rpm for 10 min, followed by filter-sterilization through 0.2 µm Ministar Syrunge Filters (Sartorius, Göttingen, Germany) and a neutralization to pH = 6.5 with 2 mol/L NaOH. The CFS were also subjected to proteolytic treatment with 10 mg/mL proteinase K (Sigma-Aldrich) at 37 °C for 2 h to ascertain the protein nature of their antagonistic activity. After proteinase K inactivation by heat (100 °C, 10 min), samples were assayed for their residual antimicrobial activity, as described above, using *P. damnosus* CECT 4797 as the indicator microorganism. Strains with antimicrobial activity in their supernatants and which were susceptible to proteinase K inactivation were considered bacteriocinogenic (Bac+) and selected for further characterization.

The antimicrobial activity of the isolates showing the highest antimicrobial effects against Gram-negative indicators was evaluated by a STOAT, as described above, but with slight modifications. Briefly, 5 µL of an overnight culture of the producer strains was spotted into M9 agar plates (1.5% *w*/*v*), and the plates were incubated aerobically at 37 °C for 24 h. Then, plates were exposed to chloroform vapors for 30 min to inactivate the producer strains. Plates were left at 37 °C to remove any traces of chloroform and then overlayed with 5 mL M9-0.8% agar seeded with 10^5^ to 10^6^ CFU/mL of an overnight culture of the following indicator strains: 6 *E. coli* strains, 6 *Salmonella* spp. strains, and 2 *Shigella* spp. strains. Plates were incubated at 37 °C for 24 h, after which zones of inhibition around the colonies were measured.

### 2.7. Whole-Genome Sequencing, Assembly, and Data Analysis

Total genomic DNA was extracted from 8 Gram-positive strains—6 *E. faecium*, 1 *L. lactis* and 1 *L. garvieae*, and 4 *E. coli* strains—by using the DNeasy Blood & Tissue Kit (Qiagen, Hilden, Germany). Purified DNA was quantified in a Qubit fluorometer (Thermo Fisher Scientific), and its quality was confirmed by agarose gel electrophoresis in 0.8% (*w*/*v*) agarose (Condalab, Madrid, Spain) gels visualized with a ChemiDoc Imaging System (BioRad). Whole-genome sequencing (WGS) of the purified DNA was performed by SeqCenter (Pittsburgh, PA, USA). Sample libraries were prepared using the Illumina DNA Prep kit and Integrated DNA Technologies (IDT) 10 bp unique dual index (UDI) indices, then sequenced on an Illumina NextSeq 2000 (Illumina, San Diego, CA, USA), producing 2 × 151 bp reads. Demultiplexing, quality control, and adapter trimming were performed with a BCL Convert v3.9.3 (Illumina). The resulting sequence reads were assembled into contigs using Unicycler v0.4.8 [28].

Bacterial species identification was confirmed by KmerFinder v.3.0.2 (https://cge.food.dtu.dk/services/KmerFinder/, accessed on 21 March 2022), which predicts bacterial species using a K-mer algorithm [29]. Annotation of the genome was performed with the Rapid Annotation Subsystem Technology (RAST) online server (http://rast.nmpdr.org/, accessed on 21 March 2022) [30]. For mining of bacteriocin and ribosomally synthesized and post-translationally modified peptides (RiPPs), the assembled genomes were analyzed under default settings in the online webserver BAGELv.4.0 (http://bagel4.molgenrug.nl/, accessed on 15 January 2023) [31] and AntiSMASH (https://antismash.secondarymetabolites.org/, accessed on 15 January 2023) [32].

The SnapGene software Version 7.0.1 (GSL Biotech, San Diego, CA, USA) was used for analysis of the bacteriocin operons. BLASTp (NCBI) and UniProt were used to confirm peptide and protein sequences, and the novelty of the putative bacteriocins was identified. The assembled genomes were also analyzed with the following bioinformatics tools: the ResFinder v.4.1 database (https://www.genomicepidemiology.org/services/, accessed on 2 February 2023) to predict the presence of acquired genes encoding antibiotic resistance and the VirulenceFinder v.2.0.3 database (https://www.genomicepidemiology.org/services/, accessed on 2 February 2023) to find genes associated with bacterial virulence factors. The presence of Mobile Genetic elements (MGE) and plasmids was evaluated with the MobileElementFinder v.3.0 (https://www.genomicepidemiology.org/services/, accessed on 2 February 2023) [33] and the PlasmidFinder https://www.genomicepidemiology.org/services/, accessed on 2 February 2023) [34], respectively. The ISfinder database (https://www-is.biotoul.fr/index.php, accessed on 22 May 2022) [35] and Prophage Hunter (https://prohunter.genomics.cn, accessed on 2 February 2023) [36] webservers were used for the identification of insertion sequences (IS) and prophages, respectively.

### 2.8. Antibiotic Susceptibility Testing

The antibiotic susceptibility of the selected isolates was assessed by using a broth microdilution test according to the European Committee on Antimicrobial Susceptibility Testing (EUCAST) guidelines. Antibiotics were selected according to the EFSA Panel on Additives and Products or Substances used in Animal Feed (FEEDAP) guidelines, and according to the guidance for the characterization of microorganisms used as feed additives or as production organisms [37]. Due to the lack of guidelines and breakpoints for *L. garvieae*, the results were interpreted according to those established for *L. lactis* [37], as the most phylogenetically related microorganism. The antibiotics tested were: ampicillin (0.25–16 mg/L), vancomycin (1–64 mg/L), gentamicin (0.5–32 mg/L), kanamycin (32–2048 mg/L), streptomycin (4–265 mg/L), erythromycin (0.25–16 mg/L), clindamycin (0.25–16 mg/L), tetracycline (0.5–32 mg/L), chloramphenicol (1–64 mg/L), and tylosin (1–64 mg/L). Briefly, all strains were grown at 32 °C for 24 h in MRS plates (Oxoid). Then, a single colony was transferred to 10 mL tubes with sterile saline solution (0.9%, *w*/*v*), adjusted to a McFarland value of 0.5 (ca. 1.5 × 10^8^ CFU/mL), and subsequently diluted 100-fold in Mueller–Hinton broth (Oxoid) (ca. 1.5 × 10^6^ cfu/mL). Then, 50 μL of the corresponding bacterial suspension was added to each well of a microtiter plate containing two-fold serial dilutions of each antibiotic (ca. 7.5 × 10^5^ cfu/mL per well). Finally, plates were incubated at 37 °C for 24 h. MICs were established as the lowest antibiotic concentration inhibiting bacterial growth and interpreted according to the cut-off values adopted by the FEEDAP guidelines. *S. aureus* ATCC 29213 (CECT 794) and *E. faecalis* ATCC 29212 (CECT 795) were used as the control microorganisms.

### 2.9. Hemolytic and Gelatinase Activities

The method previously described by [38] was used for evaluation of the hemolytic and gelatinase activities of the selected isolates. Hemolysis production was determined after overnight growth of the selected strains in MRS broth at 32 °C, further streaking onto Columbia agar plates supplemented with 5% (*v*/*v*) sheep blood (BioMérieux, Mumbai, India), and incubation at 37 °C for 24 h. The presence of clear zones of hydrolysis around the colonies indicated β-hemolysis. Gelatinase production was evaluated following streaking of the selected grown strains in MRS broth onto Todd–Hewitt agar (1.5% *w*/*v*) plates (Oxoid) supplemented with 3% (*w*/*v*) gelatin porcine skin (Oxoid). Plates were then incubated at 32 °C overnight and further maintained at 4 °C for 5 h. After this incubation, the presence of a cloudy halo around the bacterial colonies was evaluated, which is indicative of gelatin hydrolysis due to gelatinase production. *E. faecalis* P4 served as a positive control in both tests, and only isolates producing similar halos were considered positive for hemolysin production and gelatinase activity.

### 2.10. In Vitro Cell-Free Protein Synthesis (IV-CFPS) of Putative Bacteriocins Encoded by L. lactis SWD9 and Evaluation of Their Antimicrobial Activity

The plasmids pLcnP1A, pLcnP2A, pLcnP3A, and pLcnP4A were used as templates for the in vitro cell-free protein synthesis (IV-CFPS) of synthetic genes encoding the putative bacteriocins lactococcin P1A (LcnP1A), lactococcin P2A (LcnP2A), lactococcin P3A (LcnP3A), and lactococcin P4A (LcnP4A), respectively. The design and construction of the carrier plasmids encoding the synthetic genes of interest followed the criteria previously used for the construction of the PARAGEN collection of genes encoding putative bacteriocins [39]. The amino acid sequences of the putative mature bacteriocins were compared through their alignment with other closely related and previously characterized bacteriocins (garvicin Q [GarQ], lactococcin B [LcnB] and lactococcin A [LcnA]). All amino acid sequences of interest were then reverse-translated and codon-optimized for their expression by *E. coli* (www.bioinformatics.org/sms2/rev_trans.html, accessed on 5 March 2024), then placed under the control of a pUC-derived expression vector containing a T7 promoter region, a start codon (ATG), a stop codon (TAA), and a T7 terminator region. The designed gene constructs in the pUC-derived vectors (pLcnP1A, pLcnP2A, pLcnP3A, and pLcnP4A) were obtained from GeneArt (Life Technologies/Thermo Fisher Scientific).

The IV-CFPS of bacteriocins LcnP1A, LbnP2A, LcnP3A, and LcnP4A was carried out with the PURExpress In Vitro Protein Synthesis Kit (New England Biolabs, Ipswich, MA, USA) as previously described [39]. In all cases, the DNA templates were used at a final concentration of 10 ng/µL in 25 µL reactions, maintained at 37 °C for 2 h, and then placed on ice to stop the reaction. The antimicrobial activity of the IV-CFPS reactions was evaluated by using a spot-on-agar test (SOAT) [39]. Briefly, 5 μL samples from the IV-CFPS reactions were applied to the surface of BHI agar (1.5% *w*/*v*) plates previously overlaid with a BHI soft-agar (0.8% *w*/*v*) culture containing the indicator microorganisms *P. damnosus* CECT 4797 and *L. monocytogenes* CECT 4032 at approximately 10^5^ cfu/mL. The plates were then incubated at 37 °C for 24 h until zones of inhibition appeared. Similarly, 2 μL of chemically synthesized GarQ, LcnA, and LcnB at a concentration of 1 mg/mL and purity over 95%, supplied by Syngulon SA (Seraing, Belgium), was used as a positive control.

## 3. Results

### 3.1. Isolation of Antimicrobial-Producing Bacterial Isolates (API) Against Gram-Positive Indicators

**Figure 1:**
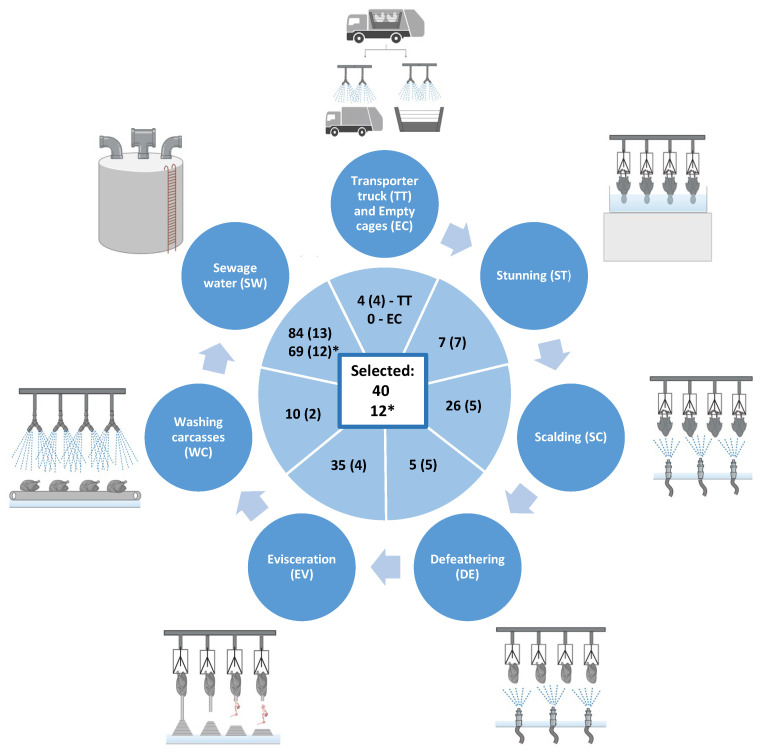
Schematic representation of the various locations from which water samples were collected in the poultry slaughterhouse. The numbers shown in the graph indicate the total isolates active against *P. damnosus* CECT4797. The figures in parentheses represent the number of bacteria subsequently selected for evaluation of their antimicrobial activity by using the stamp-on-agar test (STOAT). The asterisk denotes the isolates that showed activity against *E. coli* DH5α, with the numbers in parentheses indicating the isolates selected for their highest antimicrobial activity. The figures in the central square represent the total number of bacteria chosen for further analysis from all water samples.

Water samples from eight different sites and stages of a poultry-processing line in a slaughterhouse were collected on two non-consecutive days. Approximately 768 colonies isolated from samples (96 from each site) were screened against *P. damnosus* CECT 4797, an indicator strain with high sensitivity to most bacteriocins. A total of 171 isolates (22.3%) showed some degree of antimicrobial activity, although only 40 of them (5.2%) showed a clear halo of inhibition (Figure 1). Differences were observed in the percentage of active isolates (in terms of antimicrobial activity) recovered from the water samples tested, with a high dominance of those coming from the SW fraction (13 out of 768 [1.7%]). These 40 selected isolates were further screened for their antimicrobial activity against four different Gram-positive indicator strains: *P. damnosus* CECT 4797, *L. monocytogenes* 4032, *C. perfringens* 5A, and *S. aureus* ZTA11/00310ST (Table S1). Surprisingly, 2 out of the 40 isolates tested did not show activity against *P. damnosus* CECT 4797. However, 80% of the isolates showed some degree of antimicrobial activity against *C. perfringens* 5A, 85% against *L. monocytogenes* CECT 4032 and 55% against *S. aureus* ZTA11/00310ST. In general, the antimicrobial activity observed against the rest of the bacterial indicators was low, with the exception of isolates DFF5, SWE11, SWF2, and SWF9, with significant antimicrobial activity against *C. perfringens* 5A and *L. monocytogenes* 4032. Accordingly, 13 bacterial isolates with the highest antimicrobial activity against *P. damnosus* CECT 4797, as well as antimicrobial activity against *L. monocytogenes* 4032, *C. perfringens* 5A, and *S. aureus* ZTA11/00310ST, were selected for further analysis (Table S1).

### 3.2. Isolation of Antimicrobial-Producing Bacterial Isolates (API) Against Gram-Negative Indicators

A total of 96 colonies isolated from sewage water (SW) were screened against *E. coli* DH5α due to the high sensitivity of this strain to most of the evaluated colicins and microcins previously evaluated in our lab. From 69 isolates (71.9%) with antimicrobial activity, 12 isolates were selected for their highest antimicrobial activity and evaluated against a panel of four distinct *E. coli* strains (Table S2). From the results obtained, 6 isolates (SWB4, SWD7, SWD8, SWE2, SWF6, and SWH2) were further selected based on their high/medium antimicrobial activity against all tested indicators.

### 3.3. Taxonomic Identification and Genetic Diversity Analysis of Selected Isolates by RAPD-PCR

Out of the thirteen selected isolates with antimicrobial activity against Gram-positive bacteria, eleven were taxonomically identified as *E. faecium*, one as *L. lactis*, and one as *Lactococcus garvieae*. The phylogenetic relatedness of the eleven *E. faecium* isolates was assessed by random amplification of polymorphic DNA (RAPD). Six RADP-PCR patterns were detected, with two isolates belonging to pattern IV, four isolates to pattern V, and two isolates to pattern VI (Table S3). From the results obtained, six *E. faecium* isolates (STG2, STH9, SCH10, DEE8, SWG6, and SWB11), *L. garvieae* SWE11, and *L. lactis* SWD9 were selected for further analysis.

The six isolates selected for their antimicrobial activity against Gram-negative bacteria were taxonomically identified as *E. coli*. Based on their RAPD-PCR patterns *E. coli* SWD7 and SWD8, and *E. coli* SWE3 and SWF6 were clustered together (patterns X and XI, respectively) (Table S3). From the results obtained four *E. coli* isolates (SWB4, SWD7, SWF6 and SWH2), were selected for further analysis.

### 3.4. Antimicrobial Activity of Supernatants from the Selected Isolates

The cell-free supernatants (CFS) from the eight selected Gram-positive isolates were evaluated for their antimicrobial activity against a panel of different Gram-positive indicator strains (Table 1) using an agar diffusion test (ADT). In all cases, the antimicrobial activity against *P. damnosus* CECT 4797 was lost after proteinase K, thus confirming the proteinaceous nature of the antimicrobial compounds. Antimicrobial activity was observed against all tested indicator strains, with the exception of *S. aureus* ZTA11/00117S7, which exhibited resistance to all eight CFS. *L. garvieae* SWE11 was the most active isolate against all the indicator strains, followed by *E. faecium* SCH10, with activity against all the indicator strains except *S. aureus* ZTA11/00117S7 and *S. suis* C2969/03. While the other enterococci (STG2, STH9, DEE8, and SWG6) displayed similar antimicrobial activity, *E. faecium* SWB11 exhibited both reduced antimicrobial activity and a narrower spectrum of activity.

The antimicrobial activity of the four Gram-negative isolates selected was evaluated using the stamp-on-agar test (STOAT) and tested against a panel of different Gram-negative indicator strains (Table 2). *E. coli* SWF6 was the most active isolate, showing antimicrobial activity against all the *E. coli* indicator strains evaluated and most *Salmonella* spp. and *Shigella* spp. strains. None of the other evaluated isolates exhibited antimicrobial activity against *Salmonella* spp., and *Shigella* spp. *E. coli* SWB4 showed antimicrobial activity against all the *E. coli* indicator strains tested, while *E. coli* SWD7 and *E. coli* SWH2 showed activity against only four out of the six *E. coli* indicator strains tested.

### 3.5. Identification of Putative Bacteriocins Through in Silico Mining of the Whole Genomes of the Selected Strains

**Figure 2:**
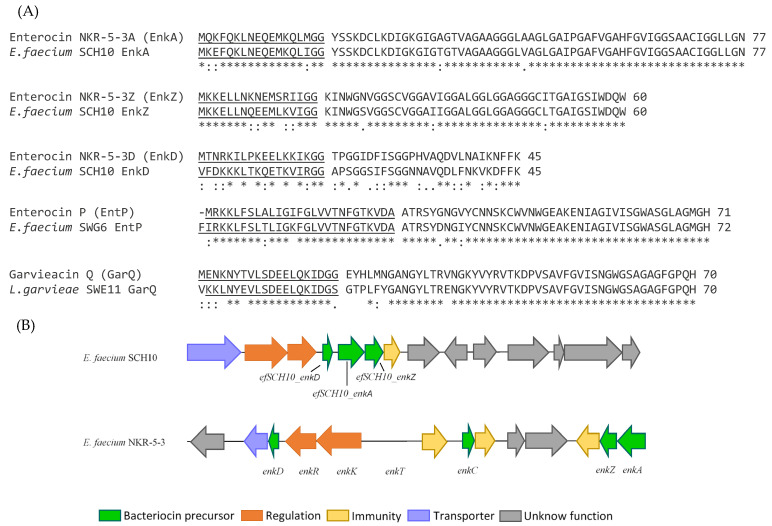
(**A**) Amino acid sequence alignment of the putative bacteriocins encoded by *E. faecium* SCH10 (EnkA-, EnkZ-, and EnkD-like bacteriocins), *E. faecium* SWG6 (EntP-like bacteriocin), and *L. garvieae* SWE11 (GarQ-like bacteriocin). The leader sequences are underlined. An asterisk (*) indicates a single fully conserved residue, a colon (:) indicates conservation within groups of residues with strongly similar properties, and a period (.) indicates conservation within groups of residues with weakly similar properties. (**B**) Genetic organization of the hypothetical bacteriocin gene cluster (BGC) identified in *E. faecium* SWE11, which includes genes encoding EnkA-, EnkZ-, and EnkD-like bacteriocins, alongside the genetic organization of the bacteriocin gene cluster from *E. faecium* NKR-3-5.

**Figure 3:**
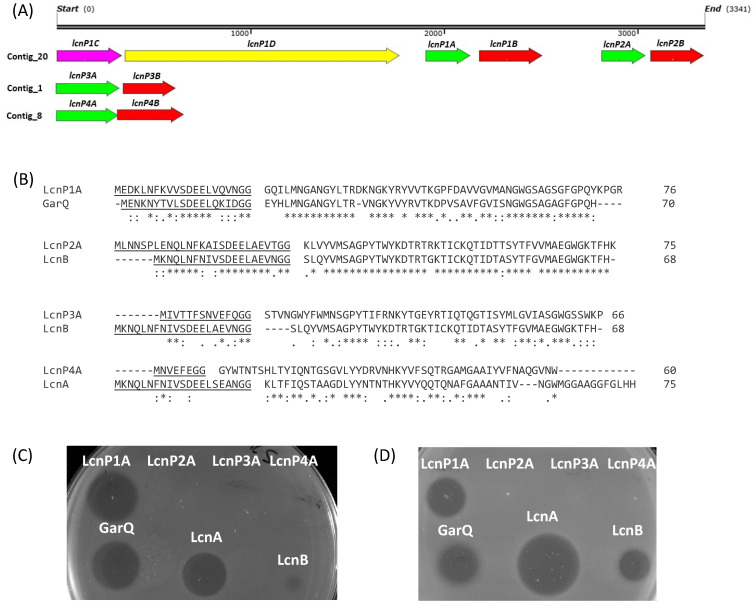
(**A**) Genetic organization of the three hypothetical bacteriocin gene clusters (BGC) identified in the *L. lactis* SWD9 genome. The structural genes for putative lactococcins are indicated by green arrows, while immunity genes are shown with red arrows. The genes *lcnP1C* and *lcnP1D* encode a putative ABC transporter and a putative transport accessory protein, respectively. (**B**) Amino acid sequence alignment of the putative bacteriocins LcnP1A, LcnP2A, LcnP3A, and LcnP4A with garvicin Q (GarQ), lactococcin A (LcnA), and lactococcin B (LcnB). The leader sequences are underlined. An asterisk (*) indicates a single fully conserved residue, a colon (:) indicates conservation within groups of residues with strongly similar properties, and a period (.) indicates conservation within groups of residues with weakly similar properties. (**C**). Antimicrobial activity against *P. damnosus* CECT 4797 and (**D**) *L. monocytogenes* CECT 4032 was evaluated using a spot-on-agar test (SOAT). This involved application to the surfaces of agar plates overlaid with a culture of the indicator microorganisms 5 µL of LcnP1A, LcnP2A, LcnP3A, and LcnP4A synthesized and produced individually by an IV-CFPS procedure, or 2 µL of chemically synthetized GarQ, LcnA, and LcnB.

Based on the results described above, twelve candidates, including eight Gram-positive and four Gram-negative strains, were selected for whole-genome sequencing (WGS) and subsequent bioinformatic analysis. Sequencing quality is detailed in Table S4. Several putative BGCs were identified by the BAGEL4 and antiSMASH7.0 servers (Table 3). Most of the BGCs identified in the genomes of the Gram-positive isolates corresponded to previously characterized class II bacteriocins. The six *E. faecium* isolates contained genes encoding the bacteriocins enterocin A (EntA), enterocin B (EntB), and enterocin X (EntXα and EntXβ). Another BGC containing genes for bacteriocins similar, but not identical, to enterocin NKR-5-3A (EnkA), enterocin NKR-5-3D (EnkD), and enterocin NKR-5-3Z (EnkZ) from *E. faecium* NKR-5-3 [40] was identified in the genome of *E. faecium* SCH10 (Figure 2A,B). *E. faecium* SWG6 also contained genes encoding a bacteriocin with 95.4% identity to enterocin P (EntP) (Figure 2A). In the genome of *L. garvieae* SWE11, a BGC was identified that included a gene encoding a bacteriocin with 93.6% identity to garvieacin Q (GarQ) (Figure 2A). Four hypothetical class IId BGCs were identified in the *L. lactis* SWD9 genome, one closely related to garvieacin Q (GarQ), two related to lactococcin B (LcnB), and the last one closely related to lactococcin A (LcnA) (Figure 3A).

Genome analysis of the *E. coli* isolates identified the presence of BGC for the production of microcin M (MccM), microcin I47 (MccI47), and microcin H47 (MccH47) in the *E. coli* SWB4 genome; microcin V (MccV) and microcin J25 (MccJ25) in *E. coli* SWF6; and colicin E7 (ColE7) in *E. coli* SWH2 (Table 3).

### 3.6. In Vitro Cell-Free Protein Synthesis (IV-CFPS) and Functionality of the Bacteriocins Identified in L. lactis SWD9

The putative bacteriocins identified in the *L. lactis* SWD9 genome were evaluated to determine their antimicrobial activity. Accordingly, all putative mature bacteriocins were produced by an in vitro cell-free protein synthesis (IV-CFPS) procedure. Four different synthetic genes were designed and cloned into pUC-derived vectors (plasmids pLcnP1A, pLcnP2A, pLcnP3A, and pLcnP4A) and used as templates for the IV-CFPS production of the bacteriocins LcnP1A, LcnP2A, LcnP3A, and LcnP4A (Figure 3B). Among the four IV-CFPS produced peptides, only LcnP1A showed antimicrobial activity against *P. damnosus* CECT 4797 and *L. monocytogenes* CECT 4032 (Figure 3C,D), two of the most sensitive strains as determined by ADT results using the CFS of *L. lactis* SWD9.

### 3.7. Genomic Characteristics of the Most Active Antimicrobial Gram-Positive Isolates

The genomes from the eight most active antimicrobial Gram-positive isolates were screened for antimicrobial resistance genes (ARG) and virulence factors to determine their suitability for probiotic use. The six *E. faecium* strains exhibited intrinsic antimicrobial resistance genes (ARGs) to gentamycicn and erythromycin, *aac(6′)-li* and *msr(C)*, respectively. Aditionally, five out of the six isolates carried the tetracycline resistance gene *tet(M)*. Notably, *E. faecium* SCH10 exhibited a total of seven different ARGs. No ARGs were detected in the *L. garvieae* SWE11 genome, while only *tet(M)* was identified in the *L. lactis* SWD9 genome (Table 4). These results were further validated with a microdilution test, which revealed that, despite the presence of *aac(6′)-li* and *msr(C)* in all the six *E. faecium* genomes, all the strains were sensitive to gentamycin and erythromycin except for *E. faecium* SCH10 and *E. faecium* SWB11, which exhibited resistance to erythromycin (Table 4 and Table S5). The functionality of *tet(M)* was validated in all of the five strains carrying this gene, which showed resistance to tetracycline. *E. faecium* SCH10 and *E. faecium* SWB11 were among the two more resistant strains, exhibiting resistance to six and five different antibiotics, respectively. While *E. faecium* SCH10 showed resistance to tylosin and *E. faecium* SWB11 showed resistance to clindamycin, streptomycin, and tylosin, no genes associated with these phenotypes were detected. Regarding the other two Gram-positive isolates, *L. garvieae* SWE11 exhibited resistance to clindamycin, despite the absence of a detected ARG for this antibiotic. In contrast, *L. lactis* showed resistance to tetracycline, thereby validating the genotypic results (Table 4 and Table S5).

Between 19 and 22 genes coding for putative virulence factors were identified in the genomes of the six *E. faecium* strains using the VirulenceFinder v.2.0.3 database (Table 4). However, no genes encoding virulence factors were detected in the *L. garvieae* SWE11 or *L. lactis* SWD9 genomes. No gelatinase or hemolytic activity was observed in any of the eight Gram-positive isolates evaluated.

Other elements, such as plasmid replicons, mobile elements, and prophages, were also analyzed. Plasmid replicons were detected in three out of six *E. faecium* isolates: strains SCH10 (n = 6), DEE8 (n = 5), and SWG6 (n = 2), as well as in *L. garvieae* SWE11 (n = 1) and *L. lactis* SWD9 (n = 1). Regarding the presence of mobile elements, all strains except *E. faecium* SWB11 carried at least one insertion sequence (IS). *E. faecium* SCH10 and *L. lactis* SWD9 each harbored four different IS, while *E. faecium* SCH10 also contained two IS and 2 transposons (Tn6009 and Tn6260). Additionally, a prophage element was identified in four out of the six *E. faecium* strains (Table 4).

## 4. Discussion

Despite the growing awareness of the serious threat posed by antimicrobial resistance (AMR), global antibiotic consumption in both human and veterinary medicine continues to rise at an alarming rate. This escalating consumption heightens the risk of AMR, particularly in the food chain, where cross-contamination between livestock and humans is a significant concern. In the context of poultry production, effluents from slaughterhouses act as reservoirs for antibiotic-resistant bacteria, facilitating the transfer of these pathogens across different environments [3]. This widespread dissemination underscores the urgent need for innovative antimicrobial strategies to mitigate the AMR spread.

This study aimed the isolation and characterization of bacteriocinogenic bacteria and their bacteriocins sourced from effluents of a poultry slaughterhouse. Over 850 bacterial isolates derived from water effluents at eight sites within the slaughterhouse were tested for their antimicrobial activity. The selection of these sites was intended to encompass all stages of the slaughterhouse process, from the reception of chickens to the water in the treatment plant. Based on the results obtained, 19 isolates showing the highest antimicrobial activity—13 against the tested Gram-positive indicators and 6 against the tested Gram-negative indicators—were selected for further evaluation of their bacteriocin production and safety characteristics.

These 19 isolates were taxonomically identified through 16S rDNA sequencing. These isolates were further discriminated by RAPD-PCR, resulting in eight Gram-positive strains, including six *E. faecium*, one *L. garvieae*, and one *L. lactis*, as well as four Gram-negative *E. coli* strains. The high prevalence of enterococci among the selected Gram-positive strains was expected, given their well-documented presence in poultry and poultry farms [41,42,43,44,45]. However, since the study aimed to identify strains with antimicrobial activity, the increased detection of enterococci in this context does not necessarily reflect their overall prevalence among all bacterial genera in the samples.

The 12 isolates selected were subjected to whole-genome sequencing (WGS) and screened for the presence of bacteriocin gene clusters (BGCs), antimicrobial resistance genes (ARGs), and virulence factors, as well as other genetic elements such as plasmids, mobile elements, and prophages. Multiple BGCs were identified in the genomes of the evaluated strains, most of which encoded previously characterized bacteriocins. A notable contribution of this study is the simultaneous evaluation of Gram-positive and Gram-negative bacteriocin producers for the identification of putative novel bacteriocins. In future studies, it would be interesting to analyze larger datasets of isolates to investigate the statistical significance of the identified BGCs or explore whether similar BGCs are prevalent across diverse bacterial populations.

All the *E. faecium* strains analyzed carried the genes coding for enterocin A (EntA), enterocin B (EntB), and enterocin X (EntX) (Table 3). The widespread distribution of the EntA structural gene within the *E. faecium* isolates has been described, even in human hospitals, in which the presence of the EntA gene was detected in 98% of 2428 *E. faecium* isolates collected over a six-year period from a single hospital system [46]. Many studies highlight the high prevalence of EntA among active *E. faecium* isolates, often noting that the presence of EntB is often linked to that of EntA [47,48,49]. Individually, both EntA and EntB exhibit strong antimicrobial activity against a wide range of foodborne pathogens and spoilage organisms. Notably, they have also been found to act synergistically, enhancing their inhibitory effects when combined [50]. This synergistic action is especially significant in controlling resistant or highly pathogenic bacterial strains, making these bacteriocins valuable for food safety applications and as potential therapeutic agents. Notably, all six *E. faecium* strains analyzed carried the genes for enterocin X in close proximity to the enterocin B genes, suggesting a possible genomic clustering of these antimicrobial peptides. This clustering may contribute to their coordinated expression or regulatory mechanisms, and it has been suggested that they may even share the same processing and/or transport system [51,52].

Additional bacteriocins similar, but not identical, to enterocin P (EntP), enterocin NKR-5-3A (EnkA), enterocin NKR-5-3Z (EnkZ), and enterocin NKR-5-3D (EnkD) have been identified in *E. faecium* SCH10 (Table 3). This finding highlights the widespread presence of multiple genes related to different bacteriocins within the enterococcal genome. However, the presence of the structural gene does not necessarily reflect the ability of the strain to synthesize the bacteriocin. Often, the BGC may harbor mutations in essential biosynthetic, processing, and transport genes, which can hinder production despite the presence of the structural gene [46,53]. Despite the antimicrobial activity observed in these six enterococcal strains, no further characterization efforts were undertaken until their safety properties could become ascertained.

Whole-genome sequencing (WGS) of *L. garvieae* SWE11 identified a BGC containing a gene encoding a bacteriocin with 94% identity to garvieacin Q (GarQ), a small, heat-stable, class II bacteriocin produced by *L. garvieae* BCC 43578 [54]. This bacteriocin exhibits strong antimicrobial activity against closely related bacteria, particularly pathogenic strains like *L. monocytogenes* and *L. garvieae*, by disrupting membrane integrity. This disruption may occur by locking the mannose-family phosphotransferase system (PTS^Man^) into a conformation that leads to the formation of a constitutively open pore [54,55]. Given the high similarity between the GarQ encoded by *L. garvieae* SWE11 and the GarQ produced by *L. garvieae* BCC 43578, no further characterization efforts were made until the probiotic potential of the producing strain could be assessed.

However, the WGS of *L. lactis* SWD9 revealed the presence of three distinct BGCs. The first cluster contains two genes that potentially code for bacteriocins, initially designated as lactococcin P1A (LcnP1A) and lactococcin P2A (LcnP2A), exhibiting 72% and 89.3% identity to GarQ and lactococcin B (LcnB), respectively. The LcnB is a small, hydrophobic, positively charged bacteriocin produced by *L. lactis* subsp. *cremoris* 9B4 [56]. The second BCG features a gene likely coding for a bacteriocin, initially termed lactococcin P3A (LcnP3A), with a 38.3% identity to LcnB. The third cluster includes another gene potentially coding for a bacteriocin, initially named lactococcin P4A (LcnP4A), with a 37% identity to lactococcin A (LcnA) (Figure 3A). The LcnA is a bacteriocin produced by *L. lactis* subsp. *cremoris* LMG 2130 that is synthesized as a 75-amino-acid precursor including a 21-amino-acid N-terminal extension [57]. GarQ, LcnA, and LcnB are all class IId, one-peptide linear bacteriocins lacking the conserved pediocin-like motif YGNGVXC [58]. The CFS of *L. lactis* SWD9 also showed activity against *C. perfringens*, a species considered a foodborne pathogen with a negative impact on broiler production [59]. To confirm the bioactivity of these four putative novel bacteriocins, an IV-CFPS protocol was used to produce the peptides individually and evaluate their antimicrobial activity against two bacterial indicators inhibited by the CFS of *L. lactis* SWD9. This IV-CFPS method has been implemented, optimized, and extensively used by our research group, enabling the rapid and efficient synthesis and determination of the antimicrobial activity of various circular and class II bacteriocins [39,60,61]. The IV-CFPS of the four hypothetical novel bacteriocins confirmed that LcnP1A exhibited inhibitory effects against the two tested indicator strains, while the other three bacteriocins did not (Figure 3C,D). Therefore, LcnP1A can be considered a novel class IId bacteriocin with demonstrated antagonistic activity against the foodborne pathogen *L. monocytogenes*. However, we cannot dismiss the possibility that the putative bacteriocins LcnP2A, LcnP3A, and LcnP4A may exhibit activity against other indicator strains, or that the genes annotated by BAGEL encode either non-functional bacteriocins or proteins with different functions.

Bioinformatic analysis of the genomes of the most antimicrobial-active *E. coli* strains revealed the presence of various BGCs of colicins and microcins. Notably, the strain *E. coli* SWF6 was the only strain exhibiting antimicrobial activity against most *E. coli*, *Salmonella*, and *Shigella* indicator strains (Table 2). Genome analysis of this strain identified the BGCs for microcin V (mccV) and microcin J25 (mccJ25) (Table 3). The activity of both bacteriocins against *Salmonella* has been previously documented, particularly that of mccJ25, whose potent inhibitory effect against *Salmonella* has been validated both by in vitro and in vivo models, highlighting its potential utility in veterinary medicine [62,63]. Since most *E. coli* isolates encoded previously described bacteriocins, no further efforts were made for the evaluation and characterization of the identified bacteriocins.

To validate the probiotic potential of the selected Gram-positive isolates, a comprehensive genomic analysis was conducted to identify antibiotic resistance and virulence genes (Table 4). All *E. faecium* strains carried the *aac(6′)-li* and *msrC* genes, which are associated with resistance to kanamycin/tobramycin and erythromycin, respectively. However, these two genes are considered intrinsic to the species and have been found in other *E. faecium* strains such as *E. faecium* SF68, a pharmaceutical probiotic with a long story of safe use [64]. Moreover, only the *E. faecium* SCH10 and SWB11 strains exhibited resistance to erythromycin, while no resistance to kanamycin was detected in any of the analyzed strains. Five out of the six tested *E. faecium* strains carried the *tet(M)* gene and showed resistance to tetracycline. The presence of this gene is relatively common among *E. faecium* strains, although is not considered intrinsic to the species. Notably, the evaluated *E. faecium* SCH10 and SWB11 strains were resistant to six different antibiotics, while the SWG6 strain was sensitive to all the antibiotics tested (Table 4). This study highlights the variability in antibiotic resistance profiles among *E. faecium* strains, even among isolates sharing the same environment. The observed differences in resistance patterns suggest that, while some strains may carry multiple resistance genes, others, like strain *E. faecium* SWG6, remain free of putative resistance genes, making them potentially safer candidates for probiotic applications.

Regarding the two lactococcal strains selected, *L. lactis* SWD9 carried the gene and exhibited phenotypic resistance to tetracycline, while *L. garvieae* SWE11 showed resistance to clindamycin, despite no antibiotic resistance gene (ARG) being identified in its genome (Table 4). The resistance of *L. lactis* SWD9 to tetracycline, particularly since it exceeds established cut-off values, indeed raises significant concerns. The exclusion of this strain from probiotic or feed additive use is warranted to prevent potential risks associated with transferring resistance genes to pathogenic bacteria. On the other hand, the resistance exhibited by *L. garvieae* SW11 to clindamycin without any ARG in its genome could suggest alternative mechanisms of resistance that are not linked to conventional ARG, such as efflux pumps or modifications of the antibiotic target. Thus, careful antibiotic susceptibility testing and genomic screening are essential to ensure the safety of these strains for their use in probiotic formulations, especially regarding their potential impact on human and animal health.

The results of the virulence factor (VF) analysis in the whole-genome-sequenced (WGS) isolates from this study indicate that initially, only the endocarditis antigen (*efaAfm*) gene and a gene associated with collagen adhesion (*acm*) were detected in all *E. faecium* genomes using the Virulence Finder database (VFDB), while none were found in the two lactococcal species. These two genes are predominant in *E. faecium*, and their roles in pathogenicity are extensively discussed [65,66]. However, in 2024, the VFDB was updated to include 27 putative virulence factors (VF) from *E. faecium* and *E. lactis* [67]. Analysis of the six *E. faecium* genomes using this updated database revealed the presence of 19 to 22 VFs across all strains, including *efaAfm* and *acm*. Notably, none of the hospital-associated virulence genes, such as *IS16*, *hyl*, *asa*-type genes, *cyl*, *gelE*, *fsr*, *sprE*, and *esp(fm)*, were detected (Table 4). Given the absence of these hospital-associated virulence genes and the observed lack of hemolytic or gelatinase activity in the strains, it is likely that the identified VFs may be involved in host–microbe interactions that are not necessarily pathogenic. Additionally, other elements, including plasmid replicons, mobile elements, and prophage elements, were identified in the analyzed genomes. However, their potential contribution to virulence remains unclear.

The isolation of *E. faecium* SWG6, which encodes multiple bacteriocins and lacks antibiotic-resistant genes, underscores its potential as a probiotic. As commensals, the enterococci are naturally found in the gastrointestinal tract, mouth, and vaginal cavity of animals and humans. The evaluation of the microbial dynamics in cheese production highlights the importance of enterococci in preserving cheese quality and heritage [68]. Notably, enterococcal strains such as *E. faecium* SF68, *E. faecium* M74, *E. faecium* Smr18, and *E. faecalis* Symbiflor are currently being utilized or proposed as probiotics for humans and livestock [64,69,70]. Further research is needed to evaluate the potential of *E. faecium* SWG6 as a protective and probiotic culture.

## 5. Conclusions

Screening bacterial isolates from poultry slaughterhouse effluents for high antimicrobial activity has led to the identification of bacteriocin-producing bacteria with antagonistic effects against significant foodborne pathogens. Whole-genome sequencing (WGS) of the isolates exhibiting the highest antimicrobial activity revealed multiple biosynthetic gene clusters (BGC) in their genomes. The use of an in vitro cell-free protein synthesis (IV-CFPS) protocol enabled the identification of a novel class IId bacteriocin, lactococcin P1A (LcnP1A), encoded by *L. lactis* SWD9. Phenotypic tests and in silico genomic profiling were performed to assess the safety of the selected isolates. The absence of antibiotic resistance, combined with a lack of virulence factors commonly associated with pathogenic *Enterococcus* species, identified *E. faecium* SWG6 as a multi-bacteriocin-encoding isolate and a promising candidate for evaluation as a probiotic. These findings promote the discovery of novel bacteriocins and safer bacteriocin-producing strains, supporting their use as part of a comprehensive approach to reduce antimicrobial resistance (AMR) in the food chain. By incorporating novel bacteriocins and novel bacteriocin-producing bacteria into food-processing operations and animal husbandry, it may be possible to curb the spread of antibiotic-resistant pathogens, ensuring safer food products and contributing to global efforts against AMR.

## Figures and Tables

**Table 1:** Antimicrobial activity of cell-free supernatants (CFS) from selected Gram-positive isolates against various indicator strains, determined using an agar diffusion test (ADT).

Indicator strains	Isolates							
	*E. faecium* STG2	*E. faecium* STH9	*E. faecium* SCH10	*E. faecium* DEE8	*E. faecium* SWG6	*E. faecium* SWB11	*L. garvieae* SWE11	*L. lactis* SWD9
*Pediococcus damnosus* CECT 4797	+++	++	+++	++	++	+	++	+++
*Clostridium perfringens* CECT 4110	+	+	+	+	+	+	+	+
*Listeria monocytogenes* CECT 4032	+	+	+	+	+	+	+	+
*Listeria grayii* CECT 931	+	+	+	+	+	+	+	+
*Listeria seeligeri* CECT 917	++	++	++	++	++	+	+	+
*Streptococcus suis* C2969/03	−	−	−	−	−	−	+	−
*Enterococcus faecium* AR1 (VRE)	+	+	+	+	+	−	+	+
*Enterococcus faecium* P7 (VRE)	++	+	++	+	+	−	++	+
*Enterococcus faecium* 714 (VRE)	+	+	+	+	+	−	++	−
*Enterococcus faecium* 720 (VRE)	++	++	++	+	++	−	+	+
*Enterococcus faecalis* 721	++	+	++	++	+	−	+	−
*Enterococcus faecalis* DBH18	−	−	+	−	+	+	+	+
*Lactococcus lactis* IL1403	+	−	+	−	−	−	++	++
*Lactococcus lactis* BB24	−	−	++	−	−	+	+	+
*Lactococcus lactis* NZ9000	−	−	++	−	−	−	++	−
*Lactococcus lactis* MG1363	+	−	++	+	−	−	++	+
*Lactococcus garvieae* 5806	+	+	++	+	+	−	++	−
*Staphylococcus aureus* ZTA11/00117ST	−	−	−	−	−	−	−	−

Antimicrobial activity determined as the diameter of the inhibition zone: (−, and white) no antimicrobial activity; (+, and light blue) inhibition zone between 5 and 10 mm; (++, and mid-blue) inhibition zone between 10 and 20 mm; and (+++, and dark blue) inhibition zone between 20 and 30 mm.

**Table 2:** Antimicrobial activity of the selected Gram-negative isolates against different indicator strains using the stamp-on-agar test (STOAT).

Indicators	Isolates			
	*E. coli* SWB4	*E. coli* SWF6	*E. coli* SWD7	*E. coli* SWH2
*Escherichia coli* DH5α	+++	+++	+++	++
*Escherichia coli* 0157:H7	+++	+++	+	−
*Escherichia coli* ZTA16/02317	+++	++	−	−
*Escherichia coli* ZTA16/01878	+++	+++	++	++
*Escherichia coli* ZTA16/01940	+++	+++	++	++
*Escherichia coli* ZTA16/01268	++	+++	−	+
*Salmonella choleraesuis* ZTA19/01344	−	−	−	−
*Salmonella paratyphi* 554	−	++	−	−
*Salmonella enteritidis* 4396	−	++	−	−
*Salmonella enteritidis* 1025	−	++	−	−
*Shigella* spp. JG024/41	−	+	−	−
*Shigella* spp. H065/02	−	−	−	−

Antimicrobial activity determined as the diameter of the inhibition zone: (−, and white) no antimicrobial activity; (+, and light green) inhibition zone between 1 and 3.6 mm; (++, and midgreen) inhibition zone between 3.6 and 6.0 mm; and (+++, and dark green) inhibition zone higher than 6 mm.

**Table 3:** Bacteriocin gene clusters (BGC) identified in the genome of the selected Gram-positive and Gram-negative isolates.

*E. faecium* STG2	*E. faecium* STH9	*E. faecium* SCH10	*E. faecium* DEE8	*E. faecium* SWG6	*E. faecium* SWB11	*L. garvieae* SWE11	*L. lactis* SWD9
Enterocin A (100)	Enterocin A (100)	Enterocin A (100)	Enterocin A (100)	Enterocin A (100)	Enterocin A (100)	Garvieacin Q (93.6)	Garvieacin Q (72.0)
Enterocin B (100)	Enterocin B (100)	Enterocin B (100)	Enterocin B (100)	Enterocin B (100)	Enterocin B (100)		Lactococcin B (89.3)
Enterocin X chain α (100)	Enterocin X chain α (100)	Enterocin X chain α (100)	Enterocin X chain α (100)	Enterocin X chain α (100)	Enterocin X chain α (100)		Lactococcin B (38.3)
Enterocin X chain β (100)	Enterocin X chain β (100)	Enterocin X chain β (100)	Enterocin X chain β (100)	Enterocin X chain β (100)	Enterocin X chain β (100)		Lactococcin A (37.0)
		Enterocin NKR-5-3 A (96.6)		Enterocin P (95.4)			
		Enterocin NKR-5-3 Z (93.0)					
		Enterocin NKR-5-3 D (52.0)					
***E. coli*** **SWB4**	***E. coli*** **SWF6**	***E. coli*** **SWD7**	***E. coli*** **SWH2**				
Microcin M (100)	Microcin V (100)	Colicin 1A (36.6)	Colicin E7 (100)				
Microcin I47 (59.5)	Microcin J25 (100)	Microcin L (97.1)					
Microcin H47 (100)							

In brackets: the percentage of identity of the amino acid sequence of the putative mature bacteriocin compared to the closest related characterized bacteriocin.

**Table 4:** List of antibiotic resistances, associated antibiotic resistance genes, virulence factors, plasmid replicons, mobile elements, and prophage elements detected in the eight sequenced Gram-positive strains. Abbreviations: GEN, gentamycin; TCY, tetracycline; ERI, erythromycin; CHL, chloramphenicol; CLI, clindamycin; STR, streptomycin; TYL, tylosin; Nf, not found; (-), when an antibiotic resistance gene has been identified (name of the antibiotic in brackets), but the strain is susceptible to that antibiotic.

Strain	Antibiotic Resistance		Virulence Factors	Plasmid Replicon Type	Mobile Elements	Prophage Elements
	Phenotype	Genotype				
*E. faecium* STG2	-	*aac(6′)-Ii* [GEN]	*bepA, ccpA, empA, empB, empC, fms13, fms14, fms15, fms17, fnm, gls20, gls33, gls2B, gls2B1, sagA, scm, orf1481, ptsD, acm, efa*	-	*ISEfm1*	*Lactob phig1e* NC_004305
	-	*msr(C)* [ERI]				
	TCY	*tet(M)*				
*E. faecium* STH9	-	*aac(6′)-Ii* [GEN]	*bepA, ccpA, empA, empB, empC, fms13, fms14, fms15, fms17, fnm, gls20, gls33, gls2B, gls2B1, sagA, scm, orf1481, ptsD, acm, efa*	-	*ISEfm1*	*Lactob phig1e* NC_004305
	-	*msr(C)* [ERI]				
	TCY	*tet(M)*				
*E. faecium* SCH10	-	*aac(6′)-Ii* [GEN]	*bepA, ccpA, empA, empB, empC, fms13, fms14, fms15, fms16, fms17, fms19, fms20, fms21, fnm, gls20, gls33, gls2B, gls2B1, sagA, scm, acm, efa*	*repUS12, repUS15, repUS43, rep1, rep2, rep18b*	*ISSsu5, ISEfa11, ISLgar5*, *IS256*	*-*
	ERI	*msr(C), erm(B)*				
	TCY	*tet(M)*				
	CHL	*cat(pC194)*				
	CLI	*erm(B)*				
	STR	*ant(6)-Ia*				
	TYL	Nf				
*E. faecium* DEE8	-	*aac(6′)-Ii* [GEN]	*bepA, ccpA, empA, empB, empC, fms13, fms14, fms15, fms17,fms21, fnm, gls20, gls33, gls2B, gls2B1, sagA, scm, acm, efa*	*repUS11a, repUS29, repUS43, rep2, rep14b*	*Tn6009, Tn6260, ISEfa11, ISEnfa4*	*Bacill BCJA1c* NC_006557
	-	*msr(C)* [ERI]				
	TCY	*tet(M)*				
	-	*cat(pC194)* [CHL]				
*E. faecium* SWG6	-	*aac(6′)-Ii* [GEN]	*bepA, ccpA, empA, empB, empC, fms13, fms14, fms15, fms17,fms20, fms21, fnm, gls20, gls33, gls2B, gls2B1, sagA, acm, efa*	*rep1, repUS15*	*ISEfa11, IS1062*	*Lactob phig1e* NC_004305
	-	*msr(C)* [ERI]				
*E. faecium* SWB11	-	*aac(6′)-Ii* [GEN]	*bepA, ccpA, empA, empB, empC, fms13, fms14, fms15, fms17, fnm, gls20, gls33, gls2B, gls2B1, sagA, scm, orf1481, ptsD, acm, efa*	-	-	-
	ERI	*msr(C)*				
	TCY	*tet(M)*				
	CLI	Nf				
	STR	Nf				
	TYL	Nf				
*L. lactis* SWD9	TCY	*tet(M)*	-	*repUS3*	*ISLll1, ISS1N, IS1068, IS-LL6*	-
*L. garvieae* SWE11	CLI	Nf	-	*repUS42*	*ISEfm1*	-

## Data Availability

The whole-genome assembly of the selected Gram-negative and Gram-positive bacteriocinogenic isolates is deposited in NCBI under the Bioproject accession number PRJNA1160053.

## Supplementary Materials

The following supporting information can be downloaded at: https://www.mdpi.com/article/10.3390/genes15121564/s1, Table S1. Antimicrobial activity of isolates against different Gram-positive indicator strains using the stamp-on-agar test (STOAT); Table S2. Antimicrobial activity of isolates against different Gram-negative and Gram-positive indicator strains using the stamp-on-agar test (STOAT); Table S3. Taxonomic identification of the selected Gram-positive and Gram-negative isolates was performed using 16S rDNA sequencing, while typing was conducted through RAPD-PCR; Table S4. Genome and assembly characteristics of the sequenced isolates; Table S5. Minimum inhibitory concentration (MIC) of different antibiotics against the eight Gram-positive isolates selected in this study.

## References

[1] Naghavi M., Vollset S.E., Ikuta K.S., Swetschinski L.R., Gray A.P., Wool E.E., Dekker D.M. (2024). Global burden of bacterial antimicrobial resistance 1990–2021: A systematic analysis with forecasts to 2050. Lancet.

[2] Baljit S., Abhijnan B., Kamna R. (2024). Antibiotics Misuse and Antimicrobial Resistance Development in Agriculture: A Global Challenge. Environ. Health.

[3] Koutsoumanis K., Allende A., Alvarez-Ordoñez A., Bolton D., Bover-Cid S., Chemaly M., Davies R., De Cesare A., Herman L., EFSA BIOHAZ Panel (EFSA Panel on Biological Hazards) (2021). Scientific Opinion on the role played by the environment in the emergence and spread of antimicrobial resistance (AMR) through the food chain. EFSA J..

[4] Klein E.Y., Van Boeckel T.P., Martinez E.M., Pant S., Gandra S., Levin S.A., Goossens H., Laxminarayan R. (2018). Global increase and geographic convergence in antibiotic consumption between 2000 and 2015. Proc. Natl. Acad. Sci. USA.

[5] Kumar D., Suchawan P., Siddhartha T. (2019). Antibiotic Usage in Poultry Production and Antimicrobial-Resistant *Salmonella* in Poultry. Food Safety in Poultry Meat Production.

[6] Muhammad J., Khan S., Su J.Q., El-Latif Hesham A., Ditta A., Nawab J., Ali A. (2020). Antibiotics in poultry manure and their associated health issues: A systematic review. J. Soils Sediments.

[7] Cong X., Krolla P., Khan U.Z., Savin M., Schwartz T. (2023). Antibiotic resistances from slaughterhouse effluents and enhanced antimicrobial blue light technology for wastewater decontamination. Environ. Sci. Pollut Res..

[8] Ding D., Wang B., Zhang X., Zhang J., Zhang H., Liu X., Gao Z., Yu Z. (2023). The spread of antibiotic resistance to humans and potential protection strategies. Ecotoxicol. Environ. Saf..

[9] Sugrue I., Ross R.P., Hill C. (2024). Bacteriocin diversity, function, discovery and application as antimicrobials. Nat. Rev. Microbiol..

[10] Mathur H., Field D., Rea M.C., Cotter P.D., Hill C., Ross R.P. (2017). Bacteriocin-Antimicrobial Synergy: A Medical and Food Perspective. Front. Microbiol..

[11] Soltani S., Hammami R., Cotter P.D., Rebuffat S., Said L.B., Gaudreau H., Bédard F., Biron E., Drider D., Fliss I. (2021). Bacteriocins as a new generation of antimicrobials: Toxicity aspects and regulations. FEMS Microbiol. Rev..

[12] Hahn-Löbmann S., Stephan A., Schulz S., Schneider T., Shaverskyi A., Tusé D., Giritch A., Gleba Y. (2019). Colicins and Salmocins—New Classes of Plant-Made Non-antibiotic Food Antibacterials. Front. Plant. Sci..

[13] Zhang J.N., Abu Zarin M., Lee C.K., Tan J.S. (2020). Application of bacteriocins in food preservation and infectious disease treatment for humans and livestock: A review. RSC Adv..

[14] Hernández-González J.C., Martínez-Tapia A., Lazcano-Hernández G., García-Pérez B.E., Castrejón-Jiménez N.S. (2021). Bacteriocins from Lactic Acid Bacteria. A Powerful Alternative as Antimicrobials, Probiotics, and Immunomodulators in Veterinary Medicine. Animals.

[15] Lambo M.T., Chang X., Liu D. (2021). The Recent Trend in the Use of Multistrain Probiotics in Livestock Production: An Overview. Animals.

[16] Reid G., Gadir A.A., Dhir R. (2019). Probiotics: Reiterating What They Are and What They Are Not. Front. Microbiol..

[17] Binda S., Hill C., Johansen E., Obis D., Pot B., Sanders M.E., Tremblay A., Ouwehand A.C. (2020). Criteria to Qualify Microorganisms as “Probiotic” in Foods and Dietary Supplements. Front. Microbiol..

[18] Khalifa A., Mohamed Ibrahim H.I. (2023). *Enterococcus faecium* from chicken feces improves chicken immune response and alleviates *Salmonella* infections: A pilot study. J. Anim. Sci..

[19] Wang W., Cai H., Zhang A., Chen Z., Chang W., Liu G., Deng X., Bryden W.L., Zheng A. (2020). *Enterococcus faecium* Modulates the Gut Microbiota of Broilers and Enhances Phosphorus Absorption and Utilization. Animals.

[20] Wu Y., Zhen W., Geng Y., Wang Z., Guo Y. (2019). Pretreatment with probiotic *Enterococcus faecium* NCIMB 11181 ameliorates necrotic enteritis-induced intestinal barrier injury in broiler chickens. Sci. Rep..

[21] Zhang H., Wang M., Jia J., Zhao J., Radebe S.M., Yu Q. (2021). The Protective Effect of *E. faecium* on *S. typhimurium* Infection Induced Damage to Intestinal Mucosa. Front. Vet. Sci..

[22] Boodhoo N., Shojadoost B., Alizadeh M., Astill J., Behboudi S., Sharif S. (2023). Effect of treatment with Lactococcus lactis NZ9000 on intestinal microbiota and mucosal immune responses against *Clostridium perfringens* in broiler chickens. Front. Microbiol..

[23] Navale V.D., Yadav R., Khilari A., Dharne M., Shanmugam D., Vamkudoth K.R. (2024). Dietary Supplementation of *Lactococcus lactis subsp. lactis* BIONCL17752 on Growth Performance and Gut Microbiota of Broiler Chickens. Probiotics Antimicrob. Proteins.

[24] Hu J., Ma L., Nie Y., Chen J., Zheng W., Wang X., Xie C., Zheng Z., Wang Z., Yang T. (2018). A Microbiota-Derived Bacteriocin Targets the Host to Confer Diarrhea Resistance in Early-Weaned Piglets. Cell Host Microbe.

[25] Sobrino O.J., Alba C., Arroyo R., Pérez I., Sariego L., Delgado S., Fernández L., de María J., Fumanal P., Fumanal A. (2021). Replacement of Metaphylactic Antimicrobial Therapy by Oral Administration of *Ligilactobacillus salivarius* MP100 in a Pig Farm. Front. Vet. Sci..

[26] Martín B., Corominas L., Garriga M., Aymerich T. (2009). Identification and tracing of *Enterococcus* spp. by RAPD-PCR in traditional fermented sausages and meat environment. J. Appl. Microbiol..

[27] Cintas L.M., Rodriguez J.M., Fernandez M.F., Sletten K., Nes I.F., Hernandez P.E., Holo H. (1995). Isolation and characterization of pediocin L50, a new bacteriocin from *Pediococcus acidilactici* with a broad inhibitory spectrum. Appl. Environ. Microbial..

[28] Larsen M., Cosentino S., Lukjancenko O., Saputra D., Rasmussen S., Hasman H., Sicheritz-Pontén T., Aarestrup F.M., Ussery D.W., Lund O. (2014). Benchmarking of methods for genomic taxonomy. J. Clin. Microbiol..

[29] Cineros J.L.B., Lund O. (2017). KmerFinderJS: A client-server method for fast species typing of bacteria over slow Internet connections. BioRxiv.

[30] Aziz R.K., Bartels D., Best A.A., DeJongh M., Disz T., Edwards R.A., Formsma K., Gerdes S., Glass E.M., Kubal M. (2008). The RAST Server: Rapid annotations using subsystems technology. BMC Genom..

[31] Van Heel A.J., de Jong A., Song C., Viel J.H., Kok J., Kuipers O.P. (2018). BAGEL4: A user-friendly web server to thoroughly mine RiPPs and bacteriocins. Nucleic Acids Res..

[32] Blin K., Shaw S., Augustijn H.E., Reitz Z.L., Biermann F., Alanjary M., Fetter A., Terlouw B.R., Metcalf W.W., Helfrich E.J.N. (2023). antiSMASH 7.0: New and improved predictions for detection, regulation, chemical structures and visualisation. Nucleic Acids Res..

[33] Belloso Daza M.V., Milani G., Cortimiglia C., Pietta E., Bassi D., Cocconcelli P.S. (2022). Genomic Insights of *Enterococcus faecium* UC7251, a Multi-Drug Resistant Strain From Ready-to-Eat Food, Highlight the Risk of Antimicrobial Resistance in the Food Chain. Front Microbiol..

[34] Mbanga J., Amoako D.G., Abia A.L.K., Allam M., Ismail A., Essack S.Y. (2021). Genomic Analysis of *Enterococcus* spp. Isolated From a Wastewater Treatment Plant and Its Associated Waters in Umgungundlovu District, South Africa. Front. Microbiol..

[35] Zhang Z., Schwartz S., Wagner L., Miller W. (2004). A Greedy Algorithm for Aligning DNA Sequences. J. Comput. Biol..

[36] Song W., Sun H.X., Zhang C., Cheng L., Peng Y., Deng Z., Wang D., Wang Y., Hu M., Liu W. (2019). Prophage Hunter: An integrative hunting tool for active prophages. Nucleic Acids Res..

[37] Rychen G., Aquilina G., Azimonti G., Bampidis V., Bastos M.L., Bories G., Chesson A., Cocconcelli P.S., Flachowsky G., EFSA Panel on Additives and Products or Substances used in Animal Feed (FEEDAP) (2018). Guidance on the characterisation of microorganisms used as feed additives or as production organisms. EFSA J..

[38] Muñoz-Atienza E., Gómez-Sala B., Araújo C., Campanero C., del Campo R., Hernández P.E., Herranz C., Cintas L.M. (2013). Antimicrobial activity, antibiotic susceptibility and virulence factors of Lactic Acid Bacteria of aquatic origin intended for use as probiotics in aquaculture. BMC Microbiol..

[39] Gabant P., Borrero J. (2019). PARAGEN 1.0: A Standardized Synthetic Gene Library for Fast Cell-Free Bacteriocin Synthesis. Front Bioeng. Biotechnol..

[40] Ishibashi N., Himeno K., Fujita K., Masuda Y., Perez R.H., Zendo T., Wilaipun P., Leelawatcharamas V., Nakayama J., Sonomoto K. (2012). Purification and Characterization of Multiple Bacteriocins and an Inducing Peptide Produced by *Enterococcus faecium* NKR-5-3 from Thai Fermented Fish. Biosci. Biotechnol. Biochem..

[41] Gong J., Forster R.J., Yu H., Chambers J.R., Wheatcroft R., Sabour P.M., Chen S. (2002). Molecular analysis of bacterial populations in the ileum of broiler chickens and comparison with bacteria in the cecum. FEMS Microbiol. Ecol..

[42] Stępień-Pyśniak D., Marek A., Banach T., Adaszek Ł., Pyzik E., Wilczyński J., Winiarczyk S. (2016). Prevalence and antibiotic resistance of *Enterococcus* strains isolated from poultry. Acta Vet. Hung..

[43] Dolka B., Gołębiewska–Kosakowska M., Krajewski K., Kwieciński P., Szubstarski J., Wilczyński J., Szeleszczuk P. (2017). Occurrence of *Enterococcus* spp. in poultry in Poland based on 2014–2015 data. Med. Weter..

[44] Mwikuma G., Kainga H., Kallu S.A., Nakajima C., Suzuki Y., Hang’ombe B.M. (2023). Determination of the Prevalence and Antimicrobial Resistance of *Enterococcus faecalis* and *Enterococcus faecium*. Associated with Poultry in Four Districts in Zambia. Antibiotics.

[45] Tedim A.P., Almeida-Santos A.C., Lanza V.F., Novais C., Coque T.M., Freitas A.R., Peixe L., from the ESCMID Study Group on Food- and Water-borne Infections (EFWISG) (2024). Bacteriocin distribution patterns in *Enterococcus faecium* and *Enterococcus lactis*: Bioinformatic analysis using a tailored genomics framework. Appl. Environ. Microbiol..

[46] Garretto A., Dawid S., Woods R. (2024). Increasing prevalence of bacteriocin carriage in a six-year hospital cohort of *E. faecium*. MedRxiv.

[47] Poeta P., Costa D., Rojo-Bezares B., Zarazaga M., Klibi N., Rodrigues J., Torres C. (2007). Detection of antimicrobial activities and bacteriocin structural genes in faecal enterococci of wild animals. Microbiol. Res..

[48] Arbulu S., Jiménez J.J., Gútiez L., Campanero C., del Campo R., Cintas L.M., Herranz C., Hernández P.E. (2016). Evaluation of bacteriocinogenic activity, safety traits and biotechnological potential of fecal lactic acid bacteria (LAB), isolated from Griffon Vultures (*Gyps fulvus* subsp. fulvus). BMC Microbiol..

[49] Strateva T., Dimov S.G., Atanasova D., Petkova V., Savov E., Mitov I. (2016). Molecular genetic study of potentially bacteriocinogenic clinical and dairy *Enterococcus* spp. isolates from Bulgaria. Ann. Microbiol..

[50] Casaus P., Nilsen T., Cintas L.M., Nes I.F., Hernández P.E., Holo H. (1997). Enterocin B, a new bacteriocin from *Enterococcus faecium* T136 which can act synergistically with enterocin A. Microbiology.

[51] Franz C.M., Worobo R.W., Quadri L.E., Schillinger U., Holzapfel W.H., Vederas J.C., Stiles M.E. (1999). Atypical genetic locus associated with constitutive production of enterocin B by *Enterococcus faecium* BFE 900. Appl. Environ. Microbiol..

[52] Hu C.B., Malaphan W., Zendo T., Nakayama J., Sonomoto K. (2010). Enterocin X, a novel two-peptide bacteriocin from *Enterococcus faecium* KU-B5, has an antibacterial spectrum entirely different from those of its component peptides. Appl. Environ. Microbiol..

[53] Sevillano E., Lafuente I., Peña N., Cintas L.M., Muñoz-Atienza E., Hernández P.E., Borrero J. (2024). Evaluation of safety and probiotic traits from a comprehensive genome-based in silico analysis of *Ligilactobacillus salivarius* P1CEA3, isolated from pigs and producer of nisin S. Foods.

[54] Tosukhowong A., Zendo T., Visessanguan W., Roytrakul S., Pumpuang L., Jaresitthikunchai J., Sonomoto K. (2012). Garvieacin Q, a novel class II bacteriocin from *Lactococcus garvieae* BCC 43578. Appl. Environ. Microbiol..

[55] Desiderato C.K., Hasenauer K.M., Reich S.J., Goldbeck O., Holivololona L., Ovchinnikov K.V., Reiter A., Oldiges M., Diep D.B., Eikmanns B.J. (2022). Garvicin Q: Characterization of biosynthesis and mode of action. Microb. Cell Fact..

[56] Venema K., Abee T., Haandrikman A.J., Leenhouts K.J., Kok J., Konings W.N., Venema G. (1993). Mode of Action of Lactococcin B, a Thiol-Activated Bacteriocin from *Lactococcus lactis*. Appl. Environ. Microbiol..

[57] Holo H., Nilssen O., Nes I.F. (1991). Lactococcin A, a New Bacteriocin from *Lactococcus lactis* subsp. *cremoris*: Isolation and Characterization of the Protein and Its Gene. J. Bacteriol..

[58] Cotter P.D., Hill C., Ross R.P. (2005). Bacteriocins: Developing innate immunity for food. Nat. Rev. Microbiol..

[59] Mora Z.V., Macías-Rodríguez M.E., Arratia-Quijada J., Gonzalez-Torres Y.S., Nuño K., Villarruel-López A. (2020). *Clostridium perfringens* as Foodborne Pathogen in Broiler Production: Pathophysiology and Potential Strategies for Controlling Necrotic Enteritis. Animals..

[60] Peña N., Bland M.J., Sevillano E., Muñoz-Atienza E., Lafuente I., Bakkoury M.E., Cintas L.M., Hernández P.E., Gabant P., Borrero J. (2022). In vitro and in vivo production and split-intein mediated ligation (SIML) of circular bacteriocins. Front. Microbiol..

[61] Lafuente I., Sevillano E., Peña N., Cuartero A., Hernández P.E., Cintas L.M., Muñoz-Atienza E., Borrero J. (2024). Production of Pumilarin and a Novel Circular Bacteriocin, Altitudin A, by *Bacillus altitudinis* ECC22, a Soil-Derived Bacteriocin Producer. Int. J. Mol. Sci..

[62] Marković K.G., Grujović M.Ž., Koraćević M.G., Nikodijević D.D., Milutinović M.G., Semedo-Lemsaddek T., Djilas M.D. (2022). Colicins and Microcins Produced by *Enterobacteriaceae*: Characterization, Mode of Action, and Putative Applications. Int. J. Environ. Res. Public Health.

[63] Baquero F., Beis K., Craik D.J., Li Y., Link A.J., Rebuffat S., Salomón R., Severinov K., Zirah S., Hegemann J.D. (2024). The pearl jubilee of microcin J25: Thirty years of research on an exceptional lasso peptide. Nat. Prod. Rep..

[64] Franz C.M.A.P., Pot B., Vizoso-Pinto M.G., Arini A., Coppolecchia R., Holzapfel W.H. (2024). An Update on the Taxonomy and Functional Properties of the Probiotic *Enterococcus faecium* SF68. Benef. Microbes..

[65] Urshev Z., Yungareva T. (2020). Initial safety evaluation of *Enterococcus faecium* LBB.E81. Biotechnol. Biotechnol. Equip..

[66] Holzapfel W., Arini A., Aeschbacher M., Coppolecchia R., Pot B. (2018). *Enterococcus faecium* SF68 as a model for efficacy and safety evaluation of pharmaceutical probiotics. Benef. Microbes.

[67] Roer L., Kaya H., Tedim A.P., Novais C., Coque T.M., Aarestrup F.M., Peixe L., Hasman H., Hammerum A.M., Freitas A.R. (2024). VirulenceFinder for *Enterococcus faecium* and *Enterococcus lactis*: An enhanced database for detection of putative virulence markers by using whole-genome sequencing data. Microbiol. Spectr..

[68] Serrano S., Ferreira M.V., Alves-Barroco C., Morais S., Barreto-Crespo M.T., Tenreiro R., Semedo-Lemsaddek T. (2024). Beyond Harmful: Exploring Biofilm Formation by Enterococci Isolated from Portuguese Traditional Cheeses. Foods.

[69] Hanchi H., Mottawea W., Sebei K., Hammami R. (2018). The genus *Enterococcus*: Between probiotic potential and safety concerns—An update. Front. Microbiol..

[70] Rashid M., Narang A., Thakur S., Kumar Jain S., Kaur S. (2023). Therapeutic and prophylactic effects of oral administration of probiotic *Enterococcus faecium* Smr18 in *Salmonella enterica*-infected mice. Gut Pathog..

