# Supplementary material for: Screening and Genomic Profiling of Antimicrobial Bacteria Sourced from Poultry Slaughterhouse Effluents: Bacteriocin Production and Safety Evaluation
Source: Genes (Basel). 2024 Dec 2;15(12):1564. doi: 10.3390/genes15121564 (PMC11675979; doi:10.3390/genes15121564)
Supplement: Supplementary file 1 [file genes-15-01564-s001.zip › genes-3337132-supplementary.pdf]

Table S1. Antimicrobial activity of isolates based on the antimicrobial activity showed against different Gram-positive (G+) indicator strains evaluated through the stamp-on-agar test (STOAT).

Origin	Strain	*P.damnosus* **CECT 4797**	*Cl. perfringens* **DICM15/00067-5A**	*L. monocytogenes* **CECT4032**	*S. aureus* **ZTA11/00310ST**
Transporter truck (TT)	TTC9	+	+	+	-
Transporter truck (TT)	TTE5	++	+	+	-
Transporter truck (TT)	TTE7	+	+	+	-
Transporter truck (TT)	TTH7	++	+	+	-
Stunning (ST)	STB7	+	+	+	+
Stunning (ST)	STD2	+	+	+	+
Stunning (ST)	STD11	+	+	+	+
Stunning (ST)	STE1	+	+	+	+
Stunning (ST)	STG2	+++	+	+	+
Stunning (ST)	STH9	+++	+	+	+
Stunning (ST)	STH12	+	+	-	-
Scalding (SC)	SCA1	++	-	+	-
Scalding (SC)	SCE1	++	-	+	-
Scalding (SC)	SCF2	++	+	+	-
Scalding (SC)	SCH2	++	-	+	-
Scalding (SC)	SCH10	+++	+	+	+
Defeathering (DE)	DEE8	+++	+	+	+
Defeathering (DE)	DEF5	+++	++	++	+
Defeathering (DE)	DEG1	++	-	-	-
Defeathering (DE)	DEG8	++	+	+	-
Defeathering (DE)	DEG11	++	+	+	-
Evisceration (EV)	EVA9	+	+	+	+
Evisceration (EV)	EVB1	+++	+	+	-
Evisceration (EV)	EVE2	++	-	-	-
Evisceration (EV)	EVE6	++	-	-	-
Washing carcasses (WC)	WCC1	+	+	+	+
Washing carcasses (WC)	WCC5	+	+	+	+
Sewage water (SW)	SWG6	++	+	+	+
Sewage water (SW)	SWD9	+++	+	+	+
Sewage water (SW)	SWD10	+	+	+	+
Sewage water (SW)	SWB11	++	+	+	+
Sewage water (SW)	SWC4	++	+	+	+
Sewage water (SW)	SWE11	+++	++	++	+
Sewage water (SW)	SWF2	+++	++	++	+
Sewage water (SW)	SWF3	+	+	+	+
Sewage water (SW)	SWF9	+++	++	++	+
Sewage water (SW)	SWG3	-	-	-	-
Sewage water (SW)	SWG4	++	+	+	-
Sewage water (SW)	SWH5	-	-	-	-
Sewage water (SW)	SWH9	+++	+	+	+

Activity was evaluated based on the diameter of the inhibition zone: (–) no antimicrobial activity; (+) inhibition zone between 1-3.5 mm; (++) inhibition zone between 3.6-6.1 mm; and (+++) between 7.6-10 mm. Strains names represent the water source (two firs letter) and the position in the 96 well plate (third letter and number). Highlighted in grey thos isolates that were selected for further analysis based on their higher antimicrobial activity.

Table S2. Antimicrobial activity of isolates based on the antimicrobial activity showed against different *E. coli* indicator strains evaluated through the stamp-on-agar test (STOAT).

Origin	Strain	DH5α	0157:H7	ZTA16/02317	ZTA16/01940
Sewage water (SW)	SWA2	++	+	+	+
Sewage water (SW)	SWB4	+++	+++	+++	+++
Sewage water (SW)	SWC3	+	+	+	+
Sewage water (SW)	SWC4	++	+	+	+
Sewage water (SW)	SWD2	++	+	+	+
Sewage water (SW)	SWD7	+++	+++	+++	+++
Sewage water (SW)	SWD8	+++	++	++	+
Sewage water (SW)	SWE1	+	+	+	+
Sewage water (SW)	SWE2	+++	++	++	+
Sewage water (SW)	SWF6	+++	+++	+++	+++
Sewage water (SW)	SWG2	++	+	+	-
Sewage water (SW)	SWH2	++	++	++	++

Activity was evaluated based on the diameter of the inhibition zone: (–) no antimicrobial activity; (+) inhibition zone between 1-3.5 mm; (++) inhibition zone between 3.6-6.1 mm; and (+++) between 7.6-10 mm. Strain names represent the water source (two firs letter) and the position in the 96 well plate (third letter and number). Highlighted in grey thos isolates that were selected for further analysis based on their higher antimicrobial activity.

Table S3. Taxonomic identification (16s rDNA sequencing) and typing group (RAPD-PCR) of the Gram-positive and Gram-negative isolates selected.

**Strain**	**Gram**	**Taxonomy identification**	**RAPD-PCR pattern**
STG2	+	*Enterococcus faecium*	I
STH9	+	*Enterococcus faecium*	II
SCH10	+	*Enterococcus faecium*	III
DEE8	+	*Enterococcus faecium*	IV
DEF5	+	*Enterococcus faecium*	IV
SWG6	+	*Enterococcus faecium*	V
SWF2	+	*Enterococcus faecium*	V
SWF9	+	*Enterococcus faecium*	V
SWH9	+	*Enterococcus faecium*	V
SWB11	+	*Enterococcus faecium*	VI
SWBC4	+	*Enterococcus faecium*	VI
SWE11	+	*Lactococcus garvieae*	VII
SWD9	+	*Lactococcus lactis*	VIII
SWB4	-	*Escherichia coli*	IX
SWD7	-	*Escherichia coli*	X
SWD8	-	*Escherichia coli*	X
SWE2	-	*Escherichia coli*	XI
SWF6	-	*Escherichia coli*	XI
SWH2	-	*Escherichia coli*	XII

Underlined those isolates that were selected for further analysis among the strains sharing the same typing group.

Table S4. Genome and assembly characteristics of the sequenced isolates.

Isolate	Size (Mb)	GC%	Contigs	No. of RNAs	No. of coding sequences	N_{50}	L_{50}
E. faecium STG2	2,553	37.5	37	55	2,453	190,512	5
E. faecium STH9	2,578	37.9	38	56	2,540	137,283	6
E. faecium SCH10	3,038	37.6	85	54	3,070	169,416	8
E. faecium DEE8	2,713	37.8	84	55	2,751	141,309	7
E. faecium SWG6	2,675	37.9	87	58	2,700	101,360	6
E. faecium SWB11	2,541	38.0	35	55	2,487	190,512	5
L. garvieae SWE11	2,080	38.3	42	55	2,165	114,710	6
L. lactis SWD9	2,544	34.9	37	59	2,590	319,977	3
E. coli SWB4	5,239	50.5	503	70	5,546	29,632	40
E. coli SWF6	4,923	50.7	71	85	4,855	283,060	5
E. coli SWD7	4,849	50.5	130	80	4,880	127,849	11
E. coli SWH2	4,835	51.0	182	79	4,768	91,737	18

Table S5. Minimum inhibitory concentration (MICs) of different antibiotics against the eight Gram-positive isolates selected in this study.

	MIC (mg/L)							MIC (mg/L)			
Antibiotic	Break point EFSA	*E. faecium* STG2	*E. faecium* STH9	*E. faecium* SCH10	*E. faecium* DEE8	*E. faecium* SWB11	*E. faecium* SWG6	Break point EFSA	*L. gaviae* SWE11	*L. lactis* SWD9	**E. faecalis* 794
Ampicilin	2	2	2	1	2	2	1	2	1	0.25	2
Vancomycin	4	<1	<1	2	<1	4	<1	4	2	<1	4
Gentamicyn	32	4-8	8	4-8	8	**32**	<1	32	8	2	16
Kannamycin	1024	64	64	<32	<1024	128	2	64	<32	<2	64
Streptomycin	128	32-64	32-64	**256**	16-64	**>512**	32	32	32	16	64
Erythromicin	4	4	2	**>16**	2	**>16**	2	1	<0.25	<0.25	2
Clindamycin	4	2	2	**>16**	<0.25	**>16**	<0.25	1	**16**	<0.25	16
Tetracyclin	4	**>32**	**>32**	**>32**	**32**	**>32**	<0.5	4	<0.5	**32**	8
Chloranphenicol	16	2	<1	**32**	4-8	4-8	4	8	<1	<1	4
Tylosine	4	<1	<1	**>64**	2-4	**>64**	2	n.r	2	<1	2

n.r: not required

EFSA: *Guidance on the characterization of microorganisms used as feed additives or as production organisms*
